# *Amicis Omnia Sunt Communia*: NF-κB Inhibition as an Alternative to Overcome Osteosarcoma Heterogeneity

**DOI:** 10.3390/ph17060734

**Published:** 2024-06-05

**Authors:** Mariana Medeiros, Sophia Guenka, David Bastos, Karla Laissa Oliveira, María Sol Brassesco

**Affiliations:** 1 Cell Biology Department, Ribeirão Preto Medical School, University of São Paulo, Avenida Bandeirantes, 3900-Vila Monte Alegre, Ribeirão Preto 14040-900, São Paulo, Brazil; marianamedeiros@usp.br; 2 Biology Department, Faculty of Philosophy, Sciences and Letters at Ribeirão Preto, University of São Paulo, Avenida Bandeirantes, 3900-Vila Monte Alegre, Ribeirão Preto 14040-900, São Paulo, Brazil; sogpdibb@usp.br (S.G.); dbastos60@usp.br (D.B.); 3 Regional Blood Center, University of São Paulo, Avenida Bandeirantes, 3900-Vila Monte Alegre, Ribeirão Preto 14051-140, São Paulo, Brazil; karlalaissa@hotmail.com

**Keywords:** osteossarcoma, heterogeneity, signaling pathways, NF-κB, common effector

## Abstract

Tumor heterogeneity poses a significant challenge in osteosarcoma (OS) treatment. In this regard, the “omics” era has constantly expanded our understanding of biomarkers and altered signaling pathways (i.e., PI3K/AKT/mTOR, WNT/β-catenin, NOTCH, SHH/GLI, among others) involved in OS pathophysiology. Despite different players and complexities, many commonalities have been described, among which the nuclear factor kappa B (NF-κB) stands out. Its altered activation is pervasive in cancer, with pleiotropic action on many disease-relevant traits. Thus, in the scope of this article, we highlight the evidence of NF-κB dysregulation in OS and its integration with other cancer-related pathways while we summarize the repertoire of compounds that have been described to interfere with its action. In silico strategies were used to demonstrate that NF-κB is closely coordinated with other commonly dysregulated signaling pathways not only by functionally interacting with several of their members but also by actively participating in the regulation of their transcription. While existing inhibitors lack selectivity or act indirectly, the therapeutic potential of targeting NF-κB is indisputable, first for its multifunctionality on most cancer hallmarks, and secondly, because, as a common downstream effector of the many dysregulated pathways influencing OS aggressiveness, it turns complex regulatory networks into a simpler picture underneath molecular heterogeneity.

## 1. The NF-κB Pathway

**Figure 1:**
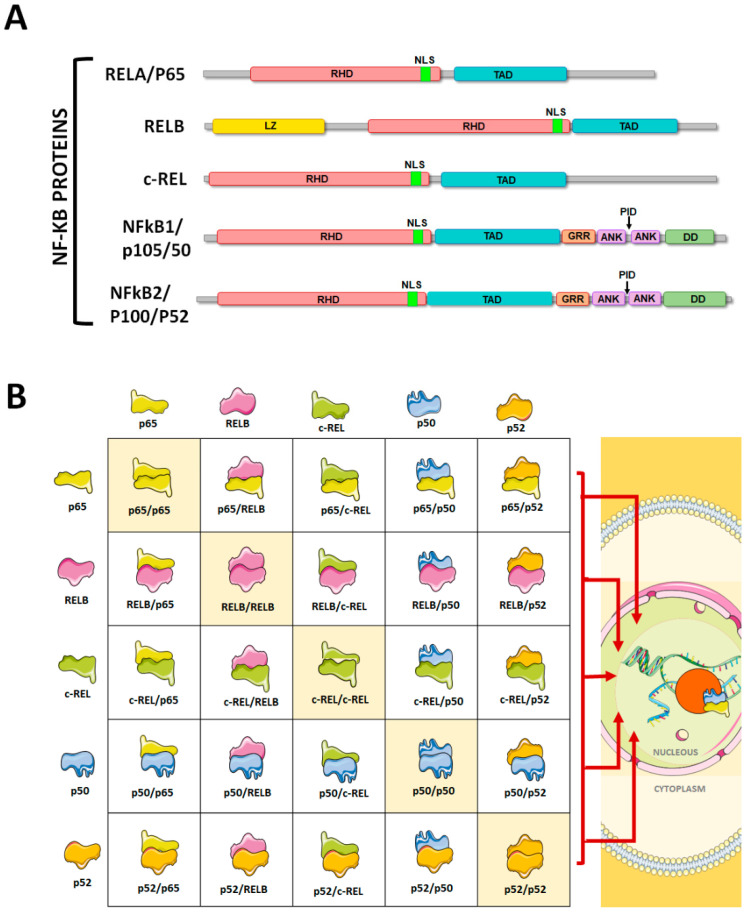
(**A**) The NF-κB family consists of 5 protein members: RELA (p65), RELB, c-REL, NF-κB1 (p105/p50), and NF-κB2 (p100/p52). Each of these proteins contains a stretch of 300 amino acids called the Rel Homology Domain (RHD), which is responsible for mediating DNA binding, dimerization, and interaction with IκB (IkappaB kinase or IKK) through the nuclear localization signal (NLS). All members also contain a transactivation domain (TAD), which mediates transcriptional induction. RELB also contains a leucine-zipper region that cooperates with the TAD. NF-κB1 and NF-κB2, which require proteolytic activation, present a glycine rich region (GRR) and multiple copies of ankyrin repeats (ANK) (within which resides the processing-inhibitory domain—PID) that are characteristic for the IκB protein family. (**B**) These proteins are found as hetero- or homodimers in the cytoplasm. Fifteen different dimers can be formed, among which RELA/p52, RELB/p52, and p52/p52 are the most commonly found in vivo. Once activated, the dimers are translocated to the cell nucleus where they exert their transcriptional function. This figure was created using Servier Medical Art templates, which are licensed under a Creative Commons Attribution 3.0 Unported License; https://smart.servier.com accessed on 10 April 2024.

The NF-κB transcription factor consists of five members of the Rel family generally found as hetero- or homodimers: RelA (p65), RelB, c-Rel, p50 (NF-κB1/p105), and p52 (NF-κB2/p100) [1]. Each of these proteins present a 300 amino acid N-terminal common domain called Rel Homology Domain (RHD), which is responsible for mediating DNA binding, dimerization, and interaction with IκB (IkappaB kinase or IKK) through the nuclear location signal (NLS) [2,3,4,5,6,7,8,9,10,11] (Figure 1A,B).

**Figure 2:**
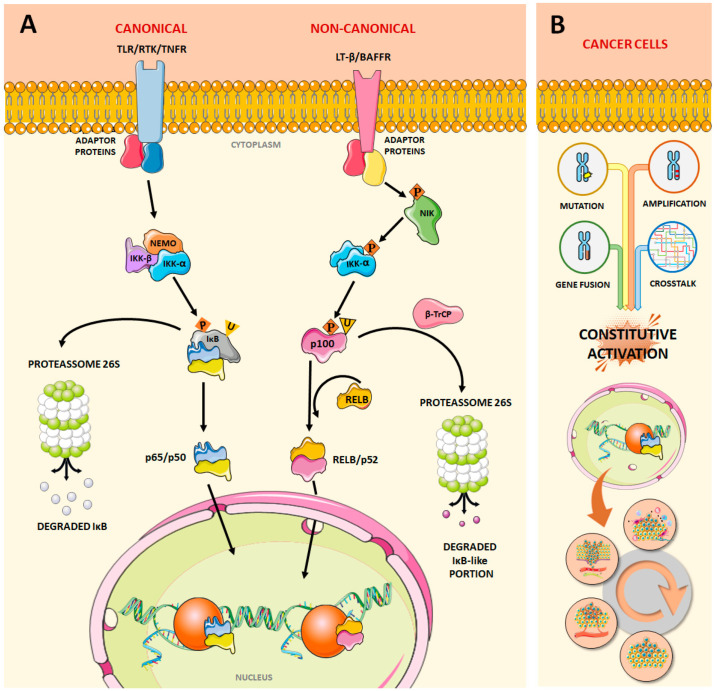
(**A**) The canonical activation of the NF-κB pathway begins with the activation of receptors located on the cell surface (toll-like receptors, TLR; receptor tyrosine kinases, RTK; and tumor necrosis factor receptors, TNFR, for instance), which then emit signals through adaptor proteins to activate the IkB kinase (IKK) complex (composed of IKK-α, the catalytic subunit IKK-β, and the regulatory subunit NEMO). Once activated, the IKK complex catalyzes the polyubiquitination and the phosphorylation of the IkBs leading to their degradation by proteasome 26S. After this, the NF-κB dimers that are released from their inhibitors and translocate to the nucleus, where they activate the transcription of many genes through binding to the consensus sequence 5′-GGGRN W YYCC-3′ (R = purine base, N = any base, W = adenine or thymine, and Y = pyrimidine base). Alternatively, the non-canonical signaling cascade begins with the activation of LT-β or BAFF receptors, which emit signals through the cytoplasm leading to the phosphorylation of the NIK protein, which, in turn, phosphorylates the IKK complex (formed by only two IKK-α subunits). Once activated, this complex phosphorylates NF-κB2/p100, in two C-terminal sites, leading to its proteolytic cleavage. This process partially degrades p100 into p52. Subsequently, the NF-κB dimer is released and translocates to the nucleus, activating gene transcription. (**B**) In cancer cells, mutations, gene amplification and gene fusions involving its subunits, or interplay with other dysregulated signaling pathways may lead to the constitutive activation of NF-κB which contributes to cancer development and progression by increasing the expression of genes associated with antiapoptosis, survival, chemo- and radioresistance, adherence, invasion, and metastasis. This figure was created using Servier Medical Art templates, which are licensed under a Creative Commons Attribution 3.0 Unported License; https://smart.servier.com accessed on 10 April 2024.

Under normal conditions, NF-κB activation occurs in response to many diverse stimuli and depends on a phosphorylation cascade initiating with the IκB proteins (IκBα, IκBβ, IκBε, IκBγ, Bcl-3, p100, and p105), which recognize the RHD domain and keep the nuclear localization signal (NLS) masked, resulting in extranuclear localization [12,13,14,15,16,17,18]. When phosphorylated, IκB proteins are degraded and expose the NLS of NF-κB dimers, resulting in nuclear translocation and gene transcription which is typically rapid and transient. This phosphorylation cascade can occur through two pathways: canonical and non-canonical (Figure 2).

The most common way to activate the canonical pathway is by the induction of tumor necrosis factor α (TNFα) [12,19]. The binding of TNFα to its toll-like receptor recruits adapter proteins responsible for activating the IκB inhibitor complex (IKK) via phosphorylation, which consists of two catalytic subunits IKKα and IKKβ and a regulatory subunit, IKKγ/NEMO kinase [20,21,22]. Once activated, IKK phosphorylates IκB, promoting its ubiquitination via a recombinant carboxyl-terminal ubiquitin hydrolase (β-TrCP) and subsequent degradation by the 26S proteasome [23,24,25]. In this way, NF-κB is translocated to the nucleus where it binds to the consensus sequence 5′-GGGRN W YYCC-3′ (R = purine base, N = any base, W = adenine or thymine, and Y = pyrimidine base) inducing gene transcription [26,27].

Alternatively, while the canonical pathway involves the RelA, c-Rel, RelB, and p50 subunits, the non-canonical pathway is responsible for processing the precursor p100 into the p52 subunit [28,29,30,31,32]. This precursor has a C-terminal processing-inhibitory domain (PID), which resides between the ankyrin repeat domain and acts as an IκB-like regulatory region. To be translocated to the nucleus in the form of a dimer with p50, p100 undergoes processing that removes the PID region. The protein responsible for initiating this process is NF-κB-inducing kinase (NIK), which promotes the activation of IKKα, which in turn phosphorylates p100, triggering its subsequent ubiquitination and degradation via the β-TrCP/26s proteasome. In this way, the newly formed dimer is translocated to the nucleus and binds to DNA at the consensus sequence [33,34,35] (Figure 2A).

## 2. NF-κB Dysregulation and Cancer

Inappropriate activation of NF-κB may mediate tumorigenesis [36]. Even though NF-κB dysregulation has been mostly ascribed to mutations in its subunits, resulting in the loss of interaction with cytoplasmatic inhibitors, other rearrangements such as amplification and gene fusions (e.g., ZFTA/RELA in supratentorial ependymomas), and crosstalk with other dysregulated signaling pathways have also been described [37,38,39,40,41,42,43,44,45,46,47,48] (Figure 2B).

Nevertheless, irrespective of the underlying mechanism, due to its pleiotropic nature, NF-κB represents an important point of convergence among different tumor hallmarks [49], affecting the expression of several survival factors, antiapoptotic genes, pro-angiogenic and pro-motility (including migration and invasion) genes, and can mediate radio- and chemoresistance mechanisms [50,51,52]. Accordingly, it greatly contributes to the appearance of more aggressive tumors and leads to worse prognoses and lower survival rates for treated patients.

Of note, this transcription factor is a known regulator in the differentiation of chondrocytes, osteoblasts, and osteocytes. Therefore, its dysregulation can initiate and/or promote sarcomagenesis [53,54,55].

### Osteosarcoma

Primary bone tumors are rare neoplasms accounting for less than 0.2% of all cancers. Osteosarcoma (OS), chondrosarcoma (ChS), and Ewing sarcoma (EWS) are the most prevalent forms [56,57], occurring in a clear bimodal age distribution, with the first peak occurring in the 10–19 age group and the second peak in elderly (>60 years-old) [58]. Their incidence remains stable worldwide and is estimated around seven individuals per million per year [59,60,61]. In addition to their rarity, these tumors show high morphological heterogeneity and variable biologic behavior [62,63], making their treatment a challenge.

OS is the most common [64], constituting 56% of all existing bone sarcomas [65]. Most patients affected with this tumor comprise children and young adults (<30 years), with a peak incidence during the “puberty growth spurt” phase [66]. However, as mentioned earlier, a second peak incidence can be observed in people over 60 years of age, especially associated with pre-existing conditions, such as Paget’s disease, for example [66,67].

The tumor is characterized by the presence of malignant mesenchymal cells, which synthesize osteoids and/or immature bone [65], being typically found in the metaphysis of long bones, mainly at the distal femur, proximal tibia, and proximal humerus [68].

OS arises mainly on the bone surface (parosteal and periosteal) and less frequently outside the bone in other tissues of mesenchymal origin (extraskeletal). Nevertheless, it usually develops in the intramedullary space with the bone membrane (periosteum) potentially rupturing during periods of accelerated growth [55,69,70]. Also, despite developing in the bone, OS is understood to be a high-grade neoplasm that presents extreme metastasis to the lung [71,72].

Its treatment typically involves neoadjuvant chemotherapy (applied before surgery) with doxorubicin, cisplatin, and high doses of methotrexate, aiming to reduce the tumor volume before resection [73,74]. Subsequently, treatment response is evaluated by determining tumor necrosis through the method described by Huvos and collaborators in 1977 [75,76,77,78]. Surgical resection consists of total tumor ablation or even limb salvage. During such treatment, chemotherapy is stopped for about 2–3 weeks and can be resumed normally thereafter [64]. Then, the last stage of treatment consists of adjuvant or postoperative chemotherapy, used with the goal of exterminating remnant neoplastic cells, improving patients’ survival [79].

Nevertheless, chemoresistance remains a significant barrier to be overcome [80]. Despite the constantly increasing number of therapeutical strategies for neoadjuvant and adjuvant treatment that has allowed the field to successfully achieve a cure in 70% of patients with localized OS, for patients diagnosed with metastatic disease at presentation, survival outcomes have remained unchanged over the past four decades, with less than 30% of patients alive after 5 years [81,82]. Radiotherapy is administered only in palliative cases, when surgical resection is not an option [83].

**Figure 3:**
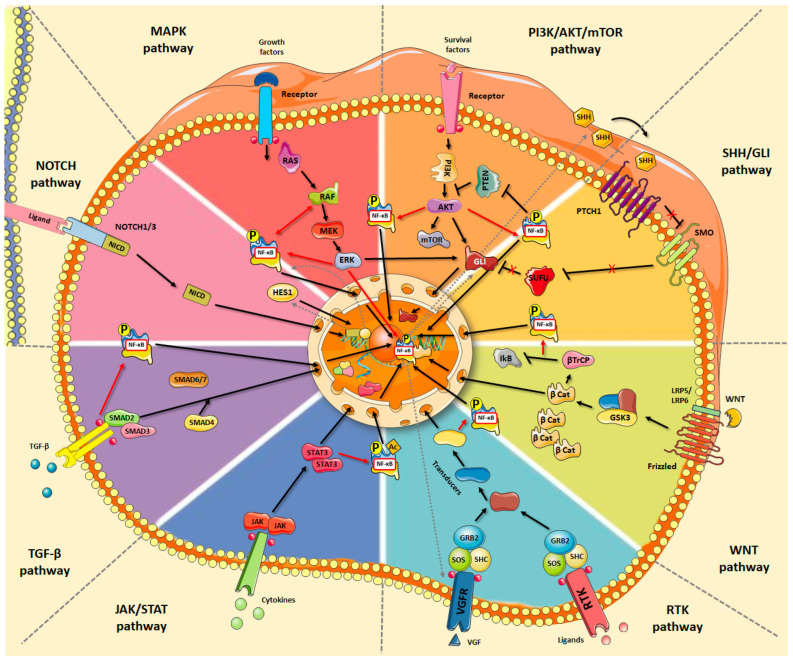
Alterations in major signaling pathways that have been identified in OS development and metastasis: PI3K/AKT/mTOR, JAK/STAT, Wnt/β-catenin, NOTCH, Hedgehog/Gli, TGF-β, MAPK, and receptor tyrosine kinases (RTKs). Despite different players and complexities, NF-κB stands out as a common downstream effector, coupling the variety of molecular cascades underneath the characteristic molecular heterogeneity of this tumor. This figure was created using Servier Medical Art templates, which are licensed under a Creative Commons Attribution 3.0 Unported License; https://smart.servier.com accessed on 11 April 2024.

From this perspective, many efforts have been made on the search for effective biomarkers that can be considered good therapeutic targets for the treatment of this tumor [84]. However, unlike other sarcomas, OS lacks recurrent genetic alterations; instead, it is highly heterogeneous with varied ploidy abnormalities, chromosomal losses and gains, and somatic DNA copy number alterations [85,86]. Inactivation of classical tumor suppressor genes such as TP53, RB1, and hyperactivation oncogenes, including MYC and MDM2, are also common [87,88]. Additionally, epigenomic, transcriptomic, proteomic, metabolomic, and functional genomic approaches have constantly expanded the number of altered signaling pathways in OS. Indeed, alterations in the major signaling pathways, such as PI3K/AKT/mTOR, JAK/STAT, WNT/β-catenin, NOTCH, Hedgehog/Gli, TGF-β, MAPK, and the receptor tyrosine kinases (RTKs) signaling pathways, have been identified in OS development and metastasis [89]. In all cases, the primary consequence of each signaling cascade is the activation of specific target genes by signal-regulated transcription factors. Of note, despite different players and complexities, many surprising and fundamental commonalities in the transcriptional mechanisms by which these pathways control the expression of their target genes have been described, among which NF-κB stands out (Figure 3). As a fact, coupling of signaling pathways enhances the functions of individual pathways and results in a more complex regulatory network [90].

The PI3K/AKT pathway is frequently hyperactivated in OS and contributes to increased proliferation and invasion, inhibition of apoptosis, angiogenesis, and chemoresistance [91,92]. Moreover, activation of this pathway has been associated with lung metastasis and poorer prognosis [93]. Constitutive Akt activity may lead to NF-κB activation. This kinase phosphorylates several substrates and downstream effectors including IkB proteins, which in turn are degraded and allow the exposure of the NLS of NF-κB dimers, their nuclear translocation, and gene transcription [94].

JAK/STAT activation has also been involved in OS development and metastasis. Even though its activation might be indirect (i.e., long non-coding RNAs), it has been demonstrated that while STAT3 signaling inhibits IKK activity in the context of a normal immune response, in tumors, STAT3 prolongs NF-κB nuclear retention through p300-mediated RelA acetylation, thereby interfering with NF-κB nuclear export [95].

Similarly, several reports showed aberrant constitutive activation of the WNT/β-catenin signaling pathway in OS tumor development and metastasis; however, current knowledge remains uncertain because of the high complexity of this pathway [96,97]. Higher β-catenin levels in OS have been associated with poor prognosis, lung metastatic dissemination [98], and stemness [99]. Spiegelman et al., showed that overexpression of β-catenin or Wnt proteins in 293T and HeLa cells increased the expression levels of βTrCP, an E3 ubiquitin ligase receptor that mediates the ubiquitination and subsequent degradation of both β-catenin and IκBα. Thus, increased βTrCP results in enhanced degradation of IκB-α and, consequently, NF-κB transactivation without affecting IKK activity [100]. Moreover, immunoprecipitation assays in murine osteoblasts have revealed that β-catenin can physically interact with the NF-κB subunits (p65 and p50) [101,102]. In line with this notion, the TCF/LEF transcription factors, which are downstream effectors of the WNT pathways and act together with β-catenin, bind to promoters of NF-κB target genes, leading to synergistic upregulation of gene expression [103]. Furthermore, the inhibition of GSK-3β (a key regulator of β-catenin) with SB216763 in OS cells eventually led to the inhibition of the NF-κB pathway and reduced the transcription of its targets [104]. Indeed, inhibition of GSK-3β with the same drug has been shown to repress IκBα phosphorylation, NF-κB (p65) nuclear translocation, and its DNA binding activity [101]. Of note, an inverse relationship was observed between (inactive) p-Ser9-GSK-3β and (active) nuclear p65 levels in OS samples, which denoted lower overall survival to OS patients. In that case, it was proposed that, when GSK-3β is impeded, IκBα is stabilized and retained in the cytoplasm enhancing apoptosis induced via chemotherapy [104].

Coactivation of NF-κB and NOTCH signaling has been previously demonstrated as well [105,106]. Notch activation can induce the expression of a large fraction of classical NF-κB gene targets in T-cell progenitors [107], and as a key player in osteogenic differentiation, bone healing, and in the development of the skeleton [108], its abnormal activation has been observed in most OS clinical specimens with a close relation with poorer prognosis [89,109]. In this regard, *NOTCH3* knockdown has been shown to deeply impair proliferation, apoptosis, and invasion in OS cells, while it reduced the number of metastatic lesions in vivo. In addition, the expression of this receptor was considered a prognostic factor correlated with metastasis and poor patient outcome. In the same context, expression of HES1, a downstream effector of NOTCH signaling, was reduced after *NOTCH3* silencing in the human OS cell line U2OS [110]. Activated NOTCH1 also induced HES1 and sustained NF-κB-signaling through NFKB1, NFKB2, RELA, and RELB. Moreover, simultaneous silencing of both receptors produced a greater drop in NF-κB activity, suggesting that NOTCH1 and NOTCH3 individually modulate NF-κB, and that both receptors are necessary for its maximal activity [111].

The role of SHH signaling in the pathogenesis of OS has also been extensively researched [81,112,113]. Evidence shows that dysregulation of this pathway occurred in both ligand-dependent and ligand-independent manners [114]. Higher expression levels of genes encoding *SHH*, *DHH*, *PTCH1*, *GLI1*, *GLI2*, and *SMO* were detected in OS cell lines [115] and were validated in the tumor samples. Moreover, associations with tumor volume and, consequently, with patient outcome were described [112]. Sustained aberrant SHH activity may result from autocrine and paracrine induction [114]. In this regard, integration between SHH signaling and NF-κB also occurs [116,117,118]. Indeed, evidence showed that NF-κB directly binds and transcriptionally activates the SHH and GLI1 promoters [119,120], supporting both ligand-dependent and ligand-independent tumor promotion.

Likewise, the TGF-β signaling pathway, which is activated in OS, affects the development of lung metastases [121] and mediates protumorigenic microenvironmental changes [122]. Its interaction with NF-κB may result from non-canonical cascades, where the activated receptor complex transmits the signal to the transcription factor [123].

Following this line, activation and autophosphorylation of RTKs results in the recruitment of a wide range of downstream signaling proteins to propagate critical cellular signaling pathways. Several mechanisms underlay the constitutive activation of RTK in human cancers including gain-of-function mutations, genomic amplification, chromosomal rearrangements, and/or autocrine activation [124]. In OS, dysregulation of many of these receptors has been associated with tumor development and metastasis. For instance, overexpression of VEGF, which leads to the autocrine activation of its receptor, is associated with a more aggressive phenotype [125,126] and was significantly associated with higher metastasis rates, higher clinical stage, and chemoresistance [127,128]. *VEGF* is a typical NF-κB target [129], which also represents an effector of VEGFR signaling [130]. Likewise, NF-κB-mediated HER2 overexpression is involved in radiation-induced repopulation in heterogeneous tumors [131]; this RTK is amplified in ~30% of patients with OS [132].

In a similar way, NF-κB is a downstream mediator of many other RTKs including the platelet-derived growth factor (PDGF) [133,134], the fibroblast growth factor receptor (FGFR) [135,136,137,138], AXL, RET, and EPHB2 [139], all of which are frequently activated in most OS tissues and cell lines, correlate with poor clinical outcomes, and have been suggested as promising therapeutic targets [140,141,142,143,144,145].

Moreover, RTK signaling generally converges to activate the RAS/RAF/MEK/ERK pathway [146]. It is well-known that activation of NF-κB represents a common outcome of the RAS/RAF/MEK/ERK signaling pathway exerting proliferative effects. ERK, for instance, can lead to its activation by phosphorylating IKK [48], and, in OS, the phosphorylation of ERK1/2 promotes invasion and metastasis. Moreover, an autocrine loop between RAF and NF-κB has also been described in some cell types [147]. Interaction with RAF has also been described to trigger NF-κB through the activation of MEKK1 and IKKβ [148]. Furthermore, RAS/RAF/MEK/ERK signaling in OS was significantly associated with immune infiltration and tumor microenvironment (TME) [149]. OS microenvironment is undeniably essential for growth and dissemination [150,151]. Beside immune evasion, stromal and other tumor-associated cells influence OS by secreting growth factors, cytokines, and exosomal and non-exosomal miRNAs that lead to metabolic reprogramming, extracellular matrix remodeling, neovascularization, drug resistance, and maintenance of the cancer stem cells phenotype [69,152].

**Figure 4:**
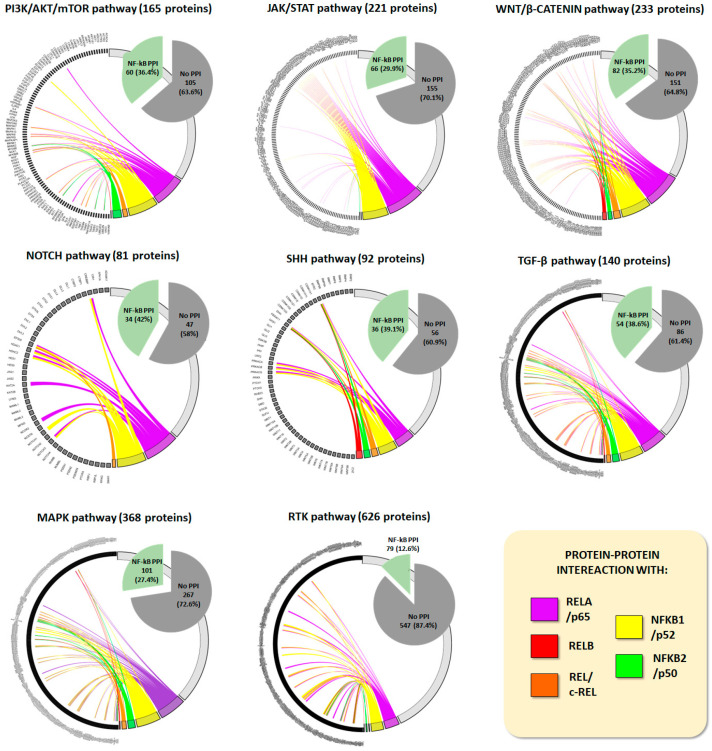
Pathways crosstalk illustration through in silico protein–protein interactions (PPI). Lists of proteins belonging to each signaling pathway were obtained though the GESEA—Gene Set Enrichment Analysis—available at https://www.gsea-msigdb.org accessed on 13 April 2024. Then, PPI between each pathway and NF-κB subunits were assessed through STRING v12 (available at https://string-db.org/ accessed on 14 April 2024). The parameters evaluated were co-occurrence, experiments, and databases, and the minimum required interaction score was 0.700, considered high. Chord plots to show individual interactions with RELA/p65, RELB, REL/c-REL, NFKB1/p52, and NFKB2/p50 were generated through the SRplot online platform for data analysis and visualization (available at http://www.bioinformatics.com.cn/srplot accessed on 14 April 2024), and the percentage of proteins involved in PPI are expressed in pie charts. As an example, there are 233 proteins involved in the WNT pathway; from these, 82 (35.2%) interact with NF-κB subunits distributed as follows: 30 proteins with RELA/p65, 8 with RELB, 10 with REL/c-REL, and 28 with NFKB2/p50. Similar patterns are seen for other pathways which, as expected, show more interactions with RELA/p65 and NFKB1/p52.

Therefore, given all the above, within the complex circuit maze underlying the molecular basis of OS, NF-κB stands out not only as a key mediator of several hallmarks of cancer biology, but also as a common denominator of intricate crosstalk with other signaling pathways crucial to shaping specific responses. This feature is highlighted with protein–protein interaction analysis showing that between 25% and 50% of proteins involved in each of the pathways cited above are functionally connected with NF-κB subunits (Figure 4–Supplemental Table S1).

Even more, the NF-κB pathway itself is dysregulated in OS [55,153,154,155,156,157,158], and, even though the mechanisms by which it plays a causative role are not yet fully understood, it clearly contributes to tumor progression and chemoresistance [159,160]. Nuclear localization of NF-κB (p65) is frequently found in OS samples [104], and higher levels of this transcription factor detected with immunohistochemical analysis denote lower survival rates [161].

**Figure 5:**
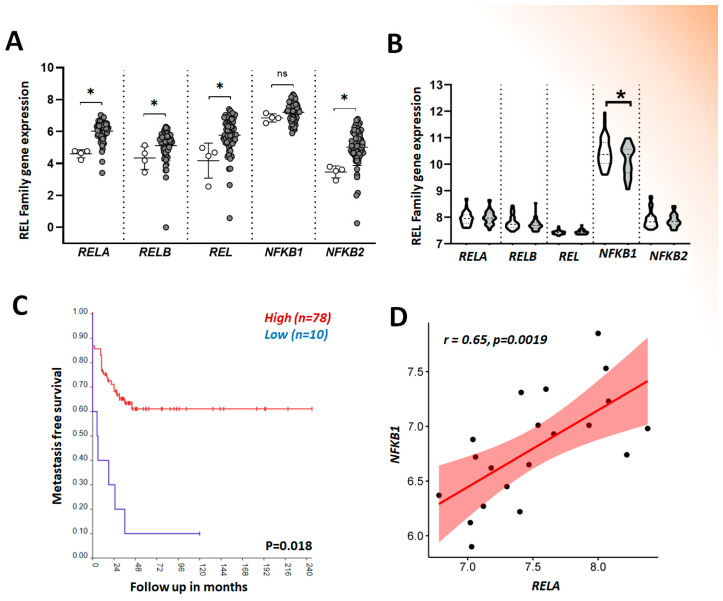
(**A**) Gene expression of *RELA*, *RELB*, *REL*, *NFKB1*, and *NFKB2* was assessed in R2 Genomics Analysis and Visualization Platform (http://r2.amc.nl accessed on 16 April 2024). Three databases were selected containing 4 control osteoblasts samples—white dots—and 61 OS samples—gray dots—(Mixed Osteosarcoma-Aqeilan-18-MAS5.0-u133p2, Mixed Osteosarcoma-Guenther-20-MAS5.0-u133a, and Tumor Osteosarcoma-Kobayashi-27-MAS5.0-u133p2; Probes: RELA 209878_s_at; RELB 205205_at; REL 235242_at; NFKB1 209239_at; NFKB2 209636_at) (ns = not significant). (**B**) Gene expression of NF-κB subunits according to the presence (gray violin) or absence (white violin) of metastasis at diagnosis using data from Mixed Osteosarcoma (Mesenchymal)-Kuijjer-127-vst-ilmnhwg6v2 also present at R2; (**C**) Kaplan–Meier curve of distant metastasis-free survival in months according to NFKB1 expression (low levels in blue, high levels in red)(generated at the R2 platform from Kuijjer’s dataset); and (**D**) Spearman correlation between RELA and NFKB1 expression in OS samples (data obtained from Guenther’s dataset). All data are expressed as log2. * = *p* < 0.05.

In this regard, an in silico analysis using gene expression of NF-κB subunits on three different OS datasets assessed at the R2 Genomics Analysis and Visualization Platform (http://r2.amc.nl accessed on 15 April 2024) (Mixed Osteosarcoma-Aqeilan-18-MAS5.0-u133p2, Mixed Osteosarcoma-Guenther-20-MAS5.0-u133a, and Tumor Osteosarcoma-Kobayashi-27-MAS5.0-u133p2) demonstrated higher levels of *RELA*, *RELB*, *REL*, and *NFKB2* compared to normal osteoblasts (*p* < 0.05) (Figure 5A). The analysis of possible associations with clinical features using the Mixed Osteosarcoma (Mesenchymal)-Kuijjer-127-vst-ilmnhwg6v2 dataset showed significantly lower levels of *NFKB1* expression in patients with metastasis at diagnosis and lower 5-year metastasis-free survival rates (Figure 5B and 5C, respectively). Also, despite a lack of differential expression between normal and OS samples, *NFKB1* showed a positive correlation with *RELA* (Figure 5D), a nonsurprising result considering that these genes encode the most abundant NF-κB heterodimer, p65/p52 [162].

**Figure 6:**
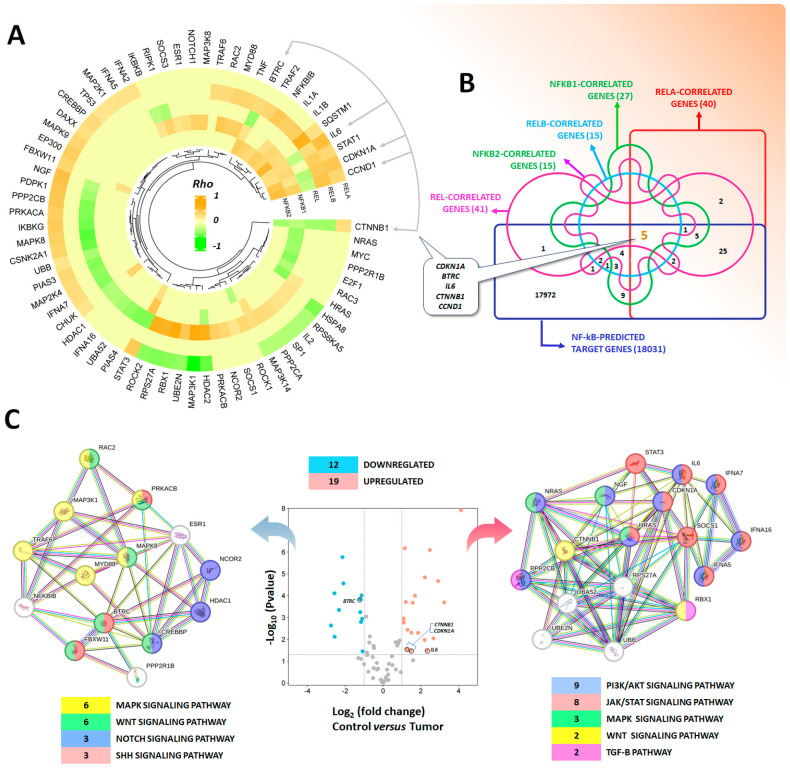
(**A**) Spearman correlation values of mRNA levels between NF-κB and pathway-associated genes depicted as a circular heatmap generated through the SRplot online platform for data analysis and visualization (available at http://www.bioinformatics.com.cn/srplot accessed on 18 April 2024). (**B**) TFLink gateway (available at https://tflink.net/ accessed on 18 April 2024) was used to investigate transcription factor–target gene interactions. A total of 61 out of 68 genes are indeed NF-κB predicted targets, of which, 5 are regulated by all NF-κB subunits and are among those with higher correlations. (**C**) Differential gene expression analysis showed 12 downregulated genes and 19 upregulated genes in OS samples and controls (Vulcano Plot generated using the Mixed Osteosarcoma (Mesenchymal)-Kuijjer-127-vst-ilmnhwg6v2 dataset). KEGG enrichment analysis through the STRING platform showed that downregulated genes belong mainly to MAPK and WNT signaling pathways, while upregulated genes are from the PI3K/AKT and JAK/STAT cascades.

Correlation between mRNA expression levels of pathway-associated genes (with the functional interaction with NF-κB shown above) was also observed, with all the 68 genes present in the Kuijjer’s dataset correlated with the expression of one or more NF-κB subunits (Figure 6A). Even though the patterns are not similar for different NF-κB subunits, analysis through the TFLink gateway (available at https://tflink.net/ accessed on 17 April 2024), which provides comprehensive and highly accurate information on transcription factor–target gene interactions, showed that 61 out of 68 genes are indeed NF-κB predicted targets (Figure 6B). Of note, five of those genes (*CDKN1A*, *BTRC*, *IL6*, *CTNNB1*, and *CCND1*) are regulated by all NF-κB subunits and are among those with higher correlation scores. Differential expression analysis showed 12 downregulated genes (mostly associated with the negative regulation of transcription: *NFKBIB*, *BTRC*, *CREBBP*, *HDAC1*, *NCOR2*, and *FBXW11*) and 19 upregulated genes [mostly associated with protein ubiquitination (*SOCS1*, *RBX1*, *RPS27A*, *UBB*, *UBA52*, and *UBE2N*), positive regulation of cell proliferation (*CTNNB1*, *NRAS*, *HRAS*, *CDKN1A*, and *STAT3*), and cell surface receptor signaling pathways (*IL6*, *IFNA5*, *IFNA7*, *IFNA16*, and *NGF*) in OS samples compared to controls. Several of these genes have already been associated with OS pathophysiology [149,163,164,165,166,167,168,169,170,171,172,173,174,175,176,177]. Moreover, KEGG functional enrichment was assessed through STRING v12 (available at https://string-db.org/ accessed on 17 April 2024) and showed that under-expressed genes belonged mainly to MAPK and WNT signaling pathways, while upregulated genes belonged to the PI3K/AKT and JAK/STAT cascades (Figure 6C), reinforcing the concept of NF-κB as a common player underneath OS establishment and progression.

Consequently, the inhibition of this transcription factor not only could result in the suppression of the proliferative and invasive capacities and chemoresistance of OS but could also improve clinical outcomes [178,179,180,181,182].

## 3. Experimental Evidence NF-κB Inhibition

As diagnosis and implementation of ideal treatment plans for a better quality of life require the integration of clinical, radiological, and histological characteristics of each malignancy, molecular markers have become key players for proper diagnosis, prognosis assessment, and prediction of treatment response. However, as the plethora of described point mutations, gene fusions, chromosomal aberrations, and epigenetic modifications increases, intra- and intertumoral heterogeneity still hampers tumor management and foment the design and subsequent development of drug candidates.

Certainly, drugs with genetically validated targets are more likely to obtain clinical approval than drugs whose targets are not supported with experimental evidence.

**Figure 7:**
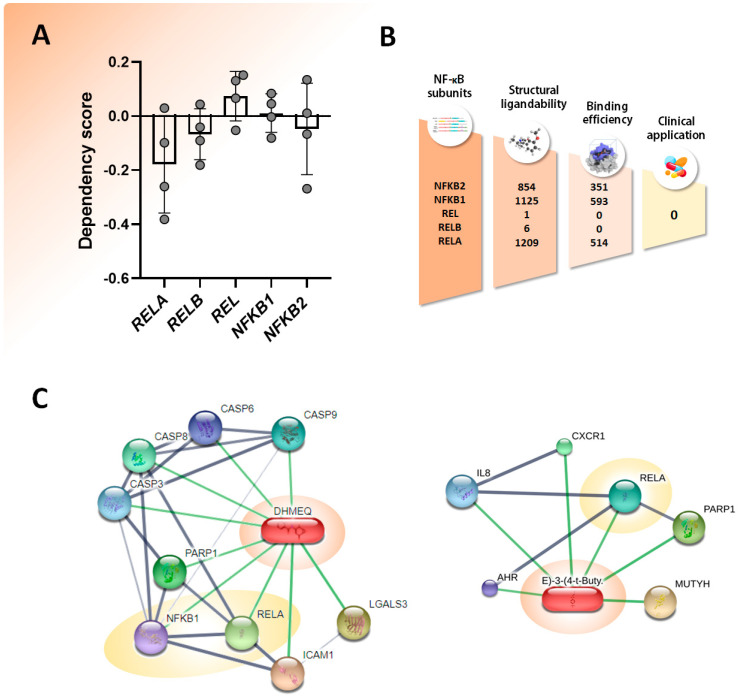
(**A**) Gene dependency on NF-κB subunits was assessed through the DepMap platform (https://depmap.org/portal/ accessed on 21 April 2024), based on CRISPR and RNAi knockout experiments on pediatric OS cell lines. Score greater than zero (>0) indicates that the cell line is not dependent, less than zero (<0) indicates that the cell line is dependent, and the closer to −1 indicates that the gene is essential for the survival of the cell line. (**B**) Schematic illustrations of NF-κB druggability identified with the CanSAR database, including the total number of compounds with predicted interaction capacity with each NF-κB subunit, binding efficiency, and the lack of proven clinical application. (**C**) Interaction networks of NF-κB inhibitors and associated binding proteins according to STITCH (available at http://stitch.embl.de accessed on 21 April 2024). From all the cited compounds with alleged activity against NF-κB, only DHMEQ and BAY 11-7085 show direct action on this transcription factor. Compounds are represented as pill-shaped nodes, while proteins are shown as spheres (smaller nodes represent proteins of unknown 3D structures). Nodes that are associated to each other are linked by an edge: thicker lines represent stronger binding affinities. Networks were constructed considering a minimum required interaction score of 0.700 and were based on associations reported in curated databases (gray lines) or on both databases and experimental/biochemical data (green lines).

In this regard, when the phenotypic relevance of each NF-κB subunit knockdown/knockout in OS cell lines is analyzed through the DepMap portal (https://depmap.org/portal/ accessed on 20 April 2024), most of them are shown to be dependent (dependency score < 0) on *RELA*, *NFKB1*, and *NFKB2* expression (Figure 7A).

Over the years, hundreds of molecules have been described as capable of interfering with the NF-κB signaling pathway. These compounds are basically divided into three types of general strategies: biomolecular inhibitors, natural products (and their derivatives), and synthetic compounds, all of which can act at different steps of the activation pathway. Within each category, these molecules can act as direct inhibitors, including IκB kinase inhibitors (IKKs), 26S proteasome inhibitors, ubiquitin-ligase complex inhibitors, and constituent NF-κB subunit inhibitors, or they can exert their activity indirectly as is the case for antioxidants [183,184]. Many NF-κB inhibitors have been extensively tested in cancer models (in vitro and in vivo) [185,186,187,188,189,190] and considered promising chemotherapeutics or chemo- or radiosensitizers. The literature shows several interesting findings regarding the use on NF-κB inhibitors for OS which are listed below and summarized in Table 1.

Amentoflavone—This is a natural biflavonoid formed from the oxidative process of two apigenin molecules commonly found in *Ginkgo biloba* [191]. Extensively researched for its wide range of activities, it notably affects the ERK/PI3K/Akt pathway and, more importantly, NF-κB signaling by inhibiting both IκBα degradation [192] and the nuclear translocation of dimers [193]. Its anticancer properties have been demonstrated in OS and various other cancer types, influencing cell proliferation, apoptosis, invasion, metastasis, autophagy, transcription, and drug-resistance. These effects are mediated through the regulation of NF-κB and related proteins such as FAS-L, TNF-alpha, and inflammatory cytokines [194]. Specifically, treatment of U2OS cells has shown impaired cell migration and invasion, evidenced by decreased expression of metastasis-associated proteins like uPA, MMP2, and MMP9 [195]. Furthermore, when combined with sorafenib, amentoflavone’s anticancer effects were enhanced, leading to cell death through extrinsic and intrinsic apoptosis signaling pathways [193]. Similar outcomes were observed in U2OS-derived xenografts treated with amentoflavone (100 mg/kg/day), where photon emission from the tumor area was three-times lower than in the control group after 15 days. Additionally, progression-associated proteins (phospho-p65, phospho-ERK, VEGF, MMP9, XIAP, and CyclinD1) were significantly reduced [196].

Andrographolide—Derived from *Andrographis paniculata*, this labdane diterpenoid exhibits notable therapeutic applications as an anti-inflammatory agent with possible antineoplastic properties [197]. Andrographolide weakens NF-κB activation by exhausting the expression levels of p65 and p50 [198]. Inhibition of p65 translocation and decreased expression levels of phosphorylated ERK and p38 have also been described after treatment [199]. In OS, this compound reduces cell viability, although the mechanism of cell death remains debated. Liu and colleagues [200] observed a dose-dependent induction of autophagy accompanied by suppression of PI3K/Akt/mTOR and enhancement of JNK signaling. Conversely, Wang and colleagues [201] demonstrated that treatment at lower concentrations induced apoptosis in U2OS cells, as evidenced by increased PARP [poly (ADP-ribose) polymerase], caspase-3, -8, and -9 cleavage. Furthermore, this treatment led to G2/M cell cycle arrest and inhibition of epithelial–mesenchymal transition, characterized by decreased levels of Snail, MMP-2, MMP-7, MMP-9, vimentin, and N-cadherin, along with increased E-cadherin expression. Additionally, andrographolide enhanced the action of pyrrolidinedithiocarbamate ammonium, another NF-κB inhibitor, and reduced the volume of orthotopic tumors and pulmonary micrometastases [202]. Consistently, andrographolide treatment showed antitumor effects on primary OS cells [201], decreasing both total and nuclear protein levels of p65 in 143B cells [202].

Bay 11-7082—Also known as Fenretinide (N-(4-hydroxyphenyl)retinamide), this is a synthetic compound that blocks IκB-α phosphorylation with an IC50 of 10 μM in HUVEC cells [203]. In OS, this vitamin A analogue induces apoptosis and impedes cell migration and invasion in vitro, while inhibiting intraosseous tumor growth in NOD/SCID mice [204]. Moreover, when combined with the PI3K inhibitor LY294002 or lithium chloride, the antiproliferative and pro-apoptotic effects were enhanced [104,204].

DHMEQ—DHMEQ (Dehydroxymethylepoxyquinomicin) is a synthetic and specific inhibitor of NF-κB, designed to block the DNA binding of p65, p50, RelB, and Rel subunits [205,206]. By doing so, it can inhibit both the canonical and non-canonical NF-κB pathway activation. DHMEQ treatment has been shown to reduce cell viability, colony formation, and the mitotic index while triggering apoptosis. Likewise, treatment reduced the migration and invasion capacities of HOS and MG-6 cell lines. When combined with standard chemotherapeutic drugs like cisplatin, doxorubicin, or methotrexate, DHMEQ showed synergistic effects, mainly in a sequential schedule [207].

Dihydroartemisinin—This semi-synthetic derivative of artemisinin, an herbal drug that has been used in traditional Chinese medicine for centuries and is commonly isolated from *Artemisia annua*, exerts its activity on NF-κB signaling by inhibiting IkBα phosphorylation and DNA binding [208,209]. This compound has demonstrated strong antitumor effects in OS cases, including decreased cell viability, proliferation, colony formation, and altered migrative capacity [210,211]. In part, these effects were attributed to caspase-dependent apoptosis and cell cycle arrest at the G2/M [212]. Similarly, treatment of orthotopic tumors can prevent OS formation and maintain intact bone structure in athymic mice treated with intragastric 20 mg/kg administration once a day for 37 days. However, the authors showed that the anticancer effects may result from inactivating Wnt/β-catenin signaling by increasing GSK3β activity and the consequent degradation of β-catenin [210]. When combined with Apatinib (VEGFR2 inhibitor), the effects were even greater, reducing cell viability, migration, and invasion in vitro, as well as reducing tumor volume in vivo [210,213]. Alternatively, it has also been demonstrated that dihydroartemisinin acts as a ROS (reactive oxygen species) generator causing mitochondrial damage, and it activated autophagy via stimulation of the ROS/Erk1/2 pathway [211,214]. Widespread changes in lipid metabolic programs in OS after treatment were also reported [213].

Dihydromyricetin—This is a natural compound extracted from *Ampelopsis grossedentata* representing a potent inhibitor of NF-κB, responsible for inhibiting IκBα phosphorylation and degradation [215]. Treatment of OS cells decreased cell viability and caused G2/M cell cycle arrest through the upregulation of p21 and induced DNA damage. Additionally, increased phosphorylation of cell cycle checkpoint proteins such as ATM, CHK2, and H2AX was also observed [216,217]. Dihydromyricetin also decreased the phosphorylation of IKKα/β, NF-κB, and IκBα in U2OS cells, while inhibited their migration and invasion ability by downregulating the expression NF-κB direct transcriptional target uPA [217]. However, there is also evidence that this flavonoid acts by interfering with p38 and the AMPKα–GSK3β–Sox2 signaling pathway [216]. Alternatively, it has also been reported that dihydromyricetin may prevent hydrogen peroxide-induced apoptosis in MG63 cells through downregulation of caspase activation and upregulation of Bcl-2 levels [218].

Ginsenoside Rh2—This bioactive compound found in *Panax ginseng* has a long-established use in Chinese traditional medicine and exhibits antiproliferative, anti-invasive, anti-metastatic, cell cycle arrest-inducing, and differentiation-promoting abilities by inhibiting NF-κB degradation [219,220]. In OS, treatment with this steroid glycoside led to reduced cell viability and increased the levels of cleaved caspase-3, caspase-8, and caspase-9, while decreasing Bcl-2 levels. Additionally, it promoted the MAPK signaling pathway while inhibiting cell migration and invasion thorough the negative regulation of MMP-2, MMP-7, MMP-9, and mesenchymal markers such as Snail, N-cadherin, and vimentin [221]. Moreover, when encapsulated in solid silica nanospheres, it reduced tumor volume and recruited immune cells in a murine OS model [222].

Isoalantolactone—This compound can be found in many medicinal plants, mainly those belonging to the Asteraceae family and other angiosperms known for producing sesquiterpene lactones (isoalantolactone, alantolactone, and 5-epoxyalantolactone) [223,224]. Isoalantolactone has shown antiproliferative effects against several cell types, including HeLa, B16F10, and MK-1 [225]. Treatment of OS cell lines (U2OS, MG63, and Saos-2) showed interesting results as well, with reduced viability and G2/M arrest. Increased apoptosis was associated with ROS generation and the dissipation of mitochondrial membrane potential. Furthermore, the study provided evidence of decreased levels of nuclear p65 in a dose-dependent manner (50% lower levels in cells treated with 40 µM compared to controls) [226].

Isoliquiritigenin—This chalcone-type flavonoid is extracted from the root or rhizome of the licorice plant *Glycyrrhiza glabra* [227]. With a broad range of pharmacological properties, it exhibits direct growth inhibitory effects in various types of cancers by blocking the nuclear translocation of NF-κB and IκBα degradation [228,229]. Also, as a broad metalloproteinase inhibitor, it caused a drastic reduction in the migration capacity of OS cells, while treatment with 0.9 mg kg^−1^ (injected intravenously into mice via the tail vein) once every 2 days for 6 days was able to reduce tumor size by about 80% in NOD/SCID mice, impeded distant organ metastasis, and prolonged the survival time (100% of the animal were alive at day 70) [230]. Other cellular processes are associated with the increased production of Bax and caspase-3 and the reduction in Bcl-2 [231,232].

β-lapachone—This naphthoquinone was originally isolated from the heartwood of the lapacho tree *Handroanthus impetiginosus* commonly found in South America [233]. With activity against several types of malignant tumors, treatment of U2-OS cells induced necrotic cell death, reductio n in mitochondrial transmembrane potential, and release of mitochondrial cytochrome c [234]. Additionally, Hori et al. (2011), demonstrated that this quinone was more efficient when combined with hyperthermia (42 °C) [235].

Licoricidin—This isoflavonoid is also extracted from the roots of the plant *G. glabra* [236,237]. Besides the known antimicrobial activities against *Helicobacter pylori*, it has shown anticancer potential [237,238]. In OS, treatment with this compound diminished viability in a dose-dependent manner. Additionally, reduced levels of p65 phosphorylation were observed both in vitro and in vivo, especially by enhancing gemcitabine-induced cytotoxicity [239].

Magnoflorine—This compound is described as an important alkaloid with a wide range of pharmacological applications that can be obtained from several members of the Ranunculaceae, Menispermaceae, and Magnoliaceae families [240]. Regarding bone tumors, magnoflorine reduced viability and invasion of MG-63 and U2OS cells in a dose-dependent manner, while it did not affect normal osteoblasts (hFOB1.19). Downregulation of p65 and IκBα phosphorylation was also correlated with increasing treatment concentrations. Additionally, enhanced sensibility of OS cells to cisplatin was also observed [241].

Matrine—This compound is the main monomer extracted from the medicinal plant *Sophora flavescens Ait*, with several pharmacological activities, including anti-inflammatory, antitumor, anti-viral, and others [242]. Initial testing in OS resulted in decreased p50 and p65 nuclear translocation and reduced levels of phosphorylated IκB-β. Cell proliferation and invasion were inhibited in a dose-dependent manner [243]. Similarly, the study by Zhou et al. (2019), showed that cotreatment with matrine significantly increased adriamycin cytotoxicity in a concentration-dependent manner. Decreased cell motility was also observed in vitro with reduced expression of MMP-9 and STAT3. In vivo, intragastric administration (50 mg/kg/day for 3 weeks) significantly reduced U2OS-derived xenografts volume [243]. Likewise, intratumoral matrine at 0.75 mg/mL for 5 weeks significantly inhibited growth of U2OS xenographic tumors [244].

Nimbolide—This chemical compound is extracted from the neem plant *Azadirachta indica* (Meliaceae family) and has shown anticancer activity through the modulation of various molecular pathways including p53, pTEN, PI3K/AKT, VEGF, Bcl-2, and NF-κB [245]. Treatment of OS cells (143B) with nimbolide reduced viability with an IC_50_ of around 4 µM [246]. Apoptosis was also observed after treatment of MG-63 and U2OS cells, as a result of endoplasmic reticulum (ER) stress, mitochondrial dysfunction, accumulation of ROS, and caspase activation. In these cells, nimbolide treatment decreased phosphorylation of IKKα/β, IκBα, and p65 as detected with luciferase activity assays [247].

Okadaic acid—This compound is considered a very potent toxin produced by dinoflagellates [248,249]. Treatment of OS cells reduced migration and induced apoptosis in a dose- and time-dependent manner, with an IC_50_ determined as 50 nM after 24 h [250].

Parthenolide—This sesquiterpene lactone of the germacranolide class, extracted from *Tanacetum parthenium* plants, acts as a covalently reactive compound that has shown selective toxicity against cancer cells at concentrations around 5–20 µM. With broad biological activity, it interferes with several pathways, albeit the most prominent and the first confirmed target was NF-κB, through alkylation of IKKβ [251] and inhibition of IκB phosphorylation [252,253]. Regarding OS, the study by D’Anneo et al. (2013) demonstrated drastic effects on viability with only 30% of cells alive after 5 h of treatment (25 µM); the DNA-binding activity of p65 also decreased rapidly (80% compared to control after 2 h of treatment), although cell death occurred in a caspase-independent manner [254]. Similar results were described by Kishida and Yoshikawa (2007) who also showed the ability of parthenolide to suppress metastasis to the lung when animals were treated soon after cell inoculation [255]. Furthermore, this compound sensitized LM7 (derived from SAOS-2) and LM8 cells to ionizing radiation [256,257].

Phloretin—This dihydrochalcone mainly found in the leaves of apple trees [258] has shown promise in cancer treatment by downregulating the expression of NF-κB, EGFR, and VEGF, and by blocking or decreasing the phosphorylation of MAP kinases among other mechanisms [259]. Huang et al. (2015) also described its effects on NF-κB signaling through the inhibition of IκB-α phosphorylation and p65 translocation to the nucleus [260]. In MG63, U2OS, and 143B cells, this inhibitor was able to reduce the viability and acted synergistically with daunomycin, 5-FU, etoposide, and methotrexate [261].

Punicalagin—This antioxidant extracted from pomegranate (*Punica granatum*) can regulate IκBα degradation and reduce p65 expression. Treatment of OS cells resulted in decreased viability and motility. Likewise, administration of punicalagin in xenograft mouse models inhibited tumor growth and diminished angiogenesis [262,263].

Raddeanin A—Extracted from the traditional Chinese herb *Anemone raddeana Regel*, this triterpenoid can inhibit p65, thereby promoting antitumor effects among other biological activities [264]. Tested as a single drug, it reduced cell viability in a panel composed of six human OS cell lines and was also responsible for reducing cell migration and invasion [265,266]. Raddeanin A also increased the cytotoxic potential of doxorubicin and downregulated MDR1 (a known target of NF-κB) in drug-resistant cells [267]. Moreover, intraperitoneal treatment with 5 mg/kg every 3 days was sufficient to reduce tumor growth in orthotopic OS models [266,267].

Sulphoraphene—Commonly found in cruciferous vegetables [268], this compound has an extensive anticancer effect by reducing p65 phosphorylation. In OS, treatment with this phytochemical reduced cell viability and colony formation in a dose-dependent manner, while impairing epithelial–mesenchymal transition (EMT). In vivo, a dose of 40 μmol/kg promoted a threefold reduction in tumor growth compared to controls after 42 days [269].

Tetramethylpyrazine—This alkaloid extracted from Chuanxiong (*Ligusticum wallichii*) exerts a variety of pharmacological effects [270] and has been shown to reduce nuclear p65 in OS. Indeed, treatment with this compound promoted G1/G0 cell cycle arrest and apoptosis by modulating cyclin D1 and BCL-2. Moreover, intraperitoneal injection (100 mg/kg doses every other day for 28 days) inhibited xenograft tumor growth with minimal effects on body weight [271].

Theabrownin—Extracted from pu-erh tea, this pigment modulates phosphorylation levels of p65 and IκBα. When tested in OS cultures, it diminished cell viability, reduced Ki67 expression, and increased cleavage of PARP and caspase-3 [272]. In addition, theabrownin inhibited the motility of U2OS cells, impairing microfilament and microtubule formation. Reinforcing this observation, treated cells expressed more E-cadherin than the mesenchymal markers vimentin, Snail-1, and Slug [273].

Thymoquinone—Naturally occurring in the seeds of *Nigella Sativa*, this compound presents well-known chemotherapeutic and chemopreventive effects, modulating NF-κB indirectly by inhibiting TNF-alpha activation [274]. Already described as a potent radiosensitizer [275], it has shown antitumor effects in OS models both as a single agent, and in combination with selenium [276], low doses of 5-FU, oxaliplatin [277], cisplatin, and methotrexate [278,279,280]. Mechanistically, in these models NF-κB inhibition increased pro-apoptotic proteins, blocked the cell cycle by promoting an increase in p21WAF1, and affected DNA metabolism proteins like g-H2AX and NBS1 [281,282]. Moreover, the administration of 6 mg/kg in tumor-bearing mice for 15 days arrested tumor growth (volume) without apparent side effects, downregulating Ki67, CD34, survivin, XIAP, and VEGF [282].

Ursolic Acid—This triterpenoid commonly used in traditional Asian medicine has been shown to inhibit the NF-κB pathway by interfering with IKKa activity, p65 phosphorylation, and DNA binding [283,284,285]. As a single agent, this compound exerted cytotoxic and antimigratory effects against several OS cell lines. The estimated half-maximum inhibitory concentration values for MG-63 were calculated to be around 11 μg/mL at 24 h and 8 μg/mL at 48 h, indicating dose- and time-dependent responses [286]. Oxidative stress and collapse of the mitochondrial membrane permeability were also observed [286,287]. These phenotypes were accompanied by the activation of ERK1/2, JNK, and MAPK signaling, and downregulation of MMP-2 and EGFR signaling. Inhibition of the JNK pathway was also reported in this tumor type [287]. In vivo, intraperitoneal administration of ursolic acid provoked a diminution of tumor growth, improving p53 expression and reducing the expression of β-catenin, NF-κB, and the phosphorylation of STAT3 [286,287]. Moreover, this antioxidant showed synergistic effects when combined with zoledronic acid [288] and cisplatin [289].

Mangostin—This compound represents a natural xanthonoid isolated from the bark and dried sap of *Garcinia mangostana* with the potential to inhibit IkBα and p65 phosphorylation [290]. Treating OS cultures promoted a reduction in cell viability and triggered apoptosis, increasing cleaved caspase-3 and PARP [291,292]. Regarding motility, mangostin inhibited invasion and migration of MG-63 cells, increasing E-cadherin and decreasing mesenchymal markers such as N-cadherin, Slug, and Snail [292]. In vivo, it promoted ER stress-mediated apoptosis caused by ROS accumulation while it restrained WNT/β-catenin signaling [291].

Genistein—This naturally occurring flavonoid acts as an antioxidant capable of downregulating NF-κB DNA binding [293]. Widely distributed in the Fabaceae family, it has been proven to exhibit good preclinical results against various types of human cancers, mainly from epithelial origin [294]. In OS models, treatment with genistein led to reduced cell viability and motility, while inducing morphological changes and differentiation denoted by increased osteocalcin [295,296,297]. Additionally, xenograft tumors derived from genistein-treated LM8 cells showed significant lower mass than controls and fewer metastases to lungs and liver [298]. When tested in combination, genistein reversed OS resistance to gemcitabine through the downregulation of NF-κB activity and the suppression of Akt [293]. Moreover, MNNG/HOS tumor-bearing mice treated daily with genistein, while receiving gemcitabine (80 mg/kg) once every other day, showed significant inhibition of tumor growth compared to controls and animals that received individual treatments [295].

Magnolol—As one of the main active components of *Magnolia officinalis*, this lignan has already shown antitumor effects through NF-κB pathway inactivation [299]. In the OS models, magnolol exposure promoted a reduction in cell viability and triggered apoptosis by inhibiting ERK/NF-κB signaling [300]. In addition, magnolol reduced colony formation, cell migration, and invasion of MG-63 cells, inducing G0/G1 cell cycle arrest and upregulation of pro-apoptosis proteins. However, a weak antiproliferative activity in normal human osteoblast cells (hFOB1.19) was also observed [301].

Bortezomib—This proteasome inhibitor, known for its ability to inhibit the NF-κB pathway, has been widely tested in OS models [302]. Its most relevant effect in this tumor type is its chemosensitizer ability. When combined with everolimus, an anti-angiogenic drug, bortezomib improved the inhibition of cell proliferation, induced cell cycle arrest, and enhanced apoptosis. Mechanistically, the combination induced higher levels of cleaved PARP, caspase-3, caspase-8, and caspase-9, while reducing the expression of c-MYC, survivin, and phospho-cyclin D1 [303]. Moreover, compared to monotherapy, the administration of everolimus and bortezomib significantly suppressed tumor growth in vivo [303,304].

Curcumin—This polyphenol derived from the turmeric rhizome of *Curcuma longa* L. is extensively described as an antitumor agent capable of constraining NF-κB pathway activation by interfering with IKK and blocking IκBα and p65 phosphorylation [305]. The literature reports numerous curcumin antiproliferative and antimigratory effects on OS with modulation of p21, Bax, Bcl-xl, Bcl2, caspase-3, PARP cleavage, cyclin D1, MMP-2, and MMP-9 expression [306,307,308,309,310,311].

However, the most interesting effects of curcumin and its analogues are their chemo- and radiosensitization properties [312,313]. For instance, combining curcumin and C6 ceramide (a type of sphingolipid that plays a role in cell differentiation, the cell cycle, cell growth, and cell death) optimized the anticancer effects of curcumin in vitro and in vivo [314]. Combinations with another plant derivative, JCTH-4 also presented synergistic effects provoking cells death by apoptosis and autophagy, in addition to an increase in ROS generation [315].

Metformin—This compound is a synthetic derivative of galegine and/or guanidine, natural products found in the herbal medicine *Galega officinalis*, and is widely used in the treatment of type 2 diabetes [316]. However, many studies discuss its anticancer capabilities, acting either directly or indirectly [317]. Accordingly, this compound has proved to be efficient against OS cell lines, including KHOS/NP, HOS, MG-63, and U2OS, and it has displayed potent in vivo antitumor effects in KHOS/NP xenografts [318]. Similarly, positive results were obtained treating 143B cells, with G2/M arrest and apoptosis attributed to ROS-dependent JNK/c-Jun activation [319].

Of note, metformin was included in a clinical trial involving children with relapsed or refractory solid and central nervous system tumors. The therapy protocol included vincristine, irinotecan, temozolomide for one cycle, and metformin given concurrently beginning in cycle 2. OS patients enrolled (n = 2) received 666 mg/m^2^/day. Grade 3 and 4 toxicities included anemia (16%), thrombocytopenia (9.6%), and neutropenia (29.8%). However, both patients presented stable diseases, reinforcing the prospects of its use in clinical setting [320].

Caffeine—This methylxanthine has long been widely used by the world population as a eugeroic or as a mild cognitive enhancer to increase alertness and attentional performance. Its antitumor effects, mainly when combined with chemotherapy, have been attributed to its potential to inhibit p65 phosphorylation [321]. In OS, simultaneous treatment with cisplatin markedly reduced cell proliferation, whereas exposure to either compound alone barely affected survival [322]. Likewise, when the effectiveness of combinations of caffeine and cisplatin were tested in cisplatin-resistant cells, the combination improved the cytotoxicity, a result not observed in cisplatin-sensitive cell lines [323]. The combination also showed positive results in vivo, both in xenografts and PDX [324,325]. In all cases, oral administration of caffeine improved the cytotoxic effects of cisplatin, reducing the tumor volume almost two times more than the administration of cisplatin alone [326]. Combined therapy led to a reduction in lung metastasis and an improvement in overall survival [327].

**Table 1:** NF-κB inhibitors tested in OS models.

Inhibitor	Target	IC_50_	In Vivo	Antiproliferation	Anti-Motility	Chemosensitizer/Synergism	Radiosensitizer	Reference
Amentoflavone	IκBα degradation and p65 translocation	50–100 μM	yes	yes	yes	yes	-	[193,196]
Andrographolide	Modification of p50	50–70 μM	yes	yes	yes	-	-	[201]
Bay 11-7085	Inhibition of IκBα	10 μM	yes	yes	yes	yes	-	[104,204]
Bortezomib	Proteasome		yes	yes	yes	yes		[303,304]
Caffeine	p65 and antioxidant	1–2.80 mM	yes	yes	yes	yes	-	[322,326]
Curcumin	IKK activety, IkBα and p65 phosphorilation	10–100 nM	yes	yes	yes	yes	-	[314,315]
DHMEQ	Inhibition of p65	12–48 μg/mL	-	yes	yes	yes	-	[206,207]
Dihydroartemisinin	IκBα degradation and DNA binding	4.6–16	yes	yes	yes	yes	-	[212]
Dihydromyricetin	Phosphorylation and degradation of IκBα	20–60 μmol/mL	yes	yes	yes	yes	-	[216]
Genistein	antioxidant	20–80 μM	yes	yes	yes	yes	-	[295,296]
Ginsenoside Rh2	NF-kB degradation	2.52–7.85 μg/mL	yes	yes	yes	yes	-	[219,220,221,222]
Isoalantolactone	p65	0–200 μM	-	yes	-	-	-	[226]
Isoliquiritigenin	p65 translocation	0–100 μM	yes	yes	yes			[230,231,232]
Lapachone	p65 phosphorilation	0–10 μM	-	yes	-	-	-	[234,235]
Licoricidin	p65	0–32 μM	yes	yes	-	yes	-	[239]
Magnoflorine	p65 phosphorilation, IkBα	5–80 μM	-	yes	yes	yes	-	[241]
Magnolol	antioxidant	25–41 μM	-	yes	yes	-	-	[300,301]
Mangostin	IkBα and p65 phosphorilation	30–40 μM	yes	yes	yes	-	-	[291,292]
Matrine	p50 and p65 translocation, IkB-β	0–1.5 mg/mL	yes	yes	yes	yes	-	[243]
Metformin	p65 phosphorylation	0–50 mM	yes	yes	yes	-	-	[316,317,318,319,320]
Nimbolide	p-IKK-β/α, p-p65	0–250 µg/mL	-	yes	yes	-	-	[246,247]
Okadaic acid	p65	0–50 nM	-	yes	yes	-	-	[248,249,250]
Parthenolide	p-65	0–100 μM	yes	yes	yes	-	yes	[257]
PHLORETIN	IκB-α phosphorylation and p65 translocation	100 μg	-	yes	-	yes	-	[261]
Punicalagin	p65	10–100 μM	yes	yes	yes	-	-	[262,263]
Raddeanin A	p65	1512–10.05 μM	yes	yes	yes	yes	-	[266,267]
Sulphoraphene	p65	40 μM	yes	yes	yes	-	-	[269]
Tetramethylpyrazine	p65	10.3, 24.7, 54.7 mg/mL	yes	yes	-	-	-	[271]
Theabrownin	p65	43.93 or 51.98 mg/mL	yes	yes	yes	-	-	[272,273]
Thymoquinone	TNF-α	17–40 μM	yes	yes	-	yes	-	[278,280,281,282]
Ursolic Acid	IKK and p65 phosphorilation	5–37 μM	yes	yes	yes	yes	-	[286,287]

## 4. Final Considerations

Despite the plethora of different NF-κB inhibitors, the majority lack selectivity or have small therapeutic indexes. For instance, an analysis on the druggability of NF-κB subunits using the CanSAR platform version 1.5.4 (an integrated knowledge base that brings together multidisciplinary data to provide useful predictions for drug discovery—https://cansarblack.icr.ac.uk/ accessed on 20 April 2024) showed that although many compounds have predicted potential for interaction, only about 45% demonstrate binding efficiency, and none of the compounds present on the platform show potential for clinical application (Figure 7B). Furthermore, interaction networks of NF-κB inhibitors and associated binding proteins according to STITCH (a search tool for known and predicted interactions between chemicals and proteins available at http://stitch.embl.de accessed on 20 April 2024) indicate that among all the compounds described above with alleged activity against NF-κB, only DHMEQ and BAY 11-7085 show direct action on this transcription factor (Figure 7C). Nevertheless, some of these substances show direct or indirect activity on many signaling pathways that ultimately prevent the activation of NF-κB. A clear example is genistein. While its cytotoxic effects are mainly attributed to the inhibition of the transcription factor, genistein also interferes with several signaling cascades that, as seen above, interplay with NF-κB: NOTCH, PI3K/Akt/mTOR, WNT/β-catenin, JAK-STAT, and RTKs pathways. Moreover, it modulates the expression of several micro-RNAs, expanding even more the range of molecular interactions to achieve the primary goal of killing cancer cells through NF-κB inhibition.

Thus, the potential to develop efficient treatments by interfering with NF-κB is undeniable, not only for its pleiotropic effects on most cancer hallmarks but also because it represents a common downstream effector of the many dysregulated pathways that influence OS aggressiveness. Deeper knowledge and understanding of how these cascades crosstalk would, over time, turn complex regulatory networks into simpler pictures underneath molecular heterogeneity, reflecting better options for controlling tumor growth and halting metastasis, which remains a major obstacle in OS treatment.

## Supplementary Materials

The following supporting information can be downloaded at: https://www.mdpi.com/article/10.3390/ph17060734/s1, Supplemental Table S1: Functional connections between NF-κB subunits and members of signaling pathways commonly dysregulated in OS. Lists of proteins belonging to each signaling pathway were obtained though the GESEA—Gene Set Enrichment Analysis—available at https://www.gsea-msigdb.org. Then, PPI between each pathway and NF-κB subunits were assessed through STRING v12 (available at https://string-db.org/ accessed on 10 April 2024).
